# Isocyanate-Free Polyurethane Coatings and Adhesives from Mono- and Di-Saccharides

**DOI:** 10.3390/polym10040402

**Published:** 2018-04-04

**Authors:** Xuedong Xi, Antonio Pizzi, Luc Delmotte

**Affiliations:** 1 LERMAB, University of Lorraine, 27 rue Philippe Seguin, 88000 Epinal, France; xuedong.xi@univ-lorraine.fr; 2 IS2M, Insti tut de Science des Matériaux de Mulhouse, CNRS LRC 7228, 15, rue Jean Starcky, BP 2488, 68057 Mulhouse, France; luc.delmotte@uha.fr

**Keywords:** polyurethanes, non-isocyanates, NIPU, adhesives, coatings, urethanes, carbonates, MALDI-TOF, ^13^C NMR, FTIR

## Abstract

Mostly biosourced non-isocyanate polyurethanes (NIPU) were prepared from mono- and disaccharides, namely glucose and sucrose, reacted with dimethyl carbonate and hexamethylene diamine. The main aim of this research was to show that NIPU can be prepared from mono- and disaccharides, this just being an initial exploratory work and its sole main aim. The oligomers obtained were detected by MALDI-ToF, CP-MAS ^13^C NMR, and FTIR spectrometries. The glucose-derived NIPU were shown to harden at a markedly lower temperature than the sucrose-derived ones and to be easier to handle and spread. The NIPU obtained were applied as wood and steel surface coatings and tested by the sessile drop test (on wood) and cross-cut test (on steel) with encouraging results. The glucose NIPU gave good surface coating results already at 103 °C, while the sucrose NIPU yielded good results only at a markedly higher temperature of hardening. The NIPU saccharide resins were also tested as thermosetting wood joint adhesives with the glucose NIPU yielding very encouraging results.

## 1. Introduction

Polyurethanes can and have already been prepared for a number of possible applications by using biosourced polyols from renewable materials [1,2,3,4,5,6,7,8]. However, polymeric isocyanates need always to be used in industry to prepare polyurethanes even if in conjunction with biosourced polyols. This has already been the case with many biosourced polyols.

Studies on alternate reaction routes to prepare non-isocyanate based polyurethanes (NIPU) do already exist. In general, oligomers terminated with five-membered cyclic carbonate groups [9] are reacted with diamines to form polyhydroxyurethanes [10]. Several studies on this type of approach do exist in the literature [11,12,13,14,15,16,17,18,19,20,21,22,23,24,25,26]. The technological barrier to the synthesis of biobased cyclic carbonates could be overcome by the chemical transformation of epoxidized vegetable oils or by the use of glycerine carbonate-based intermediates. Polyamines could also be produced from fatty diacids [27].

Dimethyl carbonate is also a good reagent and solvent that can be used for the first step of carbonation [28,29]. Indeed, it is classified as a flammable liquid with an odor similar to methanol, which has no irritating or mutagenic effects, either by contact or inhalation (indexing from Merck) [29]. 

Carboxymethylation of hydroxyl groups by dimethyl carbonate group is generally observed at temperatures around 90 °C. This is close to the boiling point of dimethyl carbonate, by bimolecular nucleophilic substitution, acyl-cleaving, in basic catalysis [29].

More recently, a few works on preparing polyurethanes without isocyanates based on tannins showed some interesting performance [30,31,32], especially for wood coating. What was more remarkable in two of them was that the small fractions of monomeric carbohydrates, mainly glucose, within the tannin extracts used for reaction, also appeared to somewhat participate to the formation of urethanes [30,32]. This was the case even when including aminated tannin as a biosourced polyamine [32]. 

This paper then deals with the initial development of the preparation of non-isocyanate-mediated urethanes starting from monosaccharides (glucose) and disaccharides (sucrose). It deals with their analysis and checking their applicability and initial potential performance as both wood surface coatings and simple thermosetting wood adhesives. Thus, the main aim of this research was to show that NIPU can be prepared from mono- and disaccharides. The present research is just an initial exploratory work, with this being its sole main aim.

## 2. Materials and Methods

### 2.1. Preparation of Isocyanate-Free Polyurethanes

The synthesis proceeded in two steps: first the glucose and the sucrose were carbonated. The carbonated glucose is called sample A1 and the carbonated sucrose sample B1. The second step consisted in reacting A1 and B1 with hexamethylene diamine to give samples A2 and B2. 

Synthesis of A1 and A2: 20 g of glucose were mixed with 13.5 g of dimethyl carbonate and 16.67 g of water, and heated to 50 °C for 40 min, and cooled to room temperature. A part of this mixture was dried at 103 °C overnight, and this was sample A1. Then, 3.88 g of hexamethylenediamine was added to 5 g of the remaining mixture in a test tube, mixed well, and then dried overnight at 103 °C. This sample was sample A2.

Synthesis of B1 and B2: Sucrose was used to instead glucose to prepare the B1, B2 under the same set of reaction conditions as A1 and A2.

The method and procedure employed are the same use for the preparation of urethanes from hydrolysable [30] and condensed tannins [31].

For the thermomechanical analysis (TMA) the samples of A1, A2, B1, and B2 were prepared as follows: glucose mixed with dimethyl carbonate and water was heated to 50 °C for 40 min and cooled at the room temperature (named A1). Hexamethylene diamine was then added to the mixture and heated to 90 °C for 30 min, then cooled to room temperature (named A2).

The sucrose mixed with dimethyl carbonate and water, heated to 50 °C for 40 min, and cooled at the room temperature, was named B1. Hexamethylene diamine was then added to the mixture and heated to 90 °C for 120 min (because the reaction was slower a longer reaction time was used), then cooled to room temperature (named B2).

It must be clearly pointed out that A1 ad B1 are just intermediate products of the two-step reaction process. It is for this reason that they have not been tested either for bonding or for coatings.

### 2.2. MALDI-TOF Analysis

Samples for matrix-assisted laser desorption ionization time-of-flight (MALDI-TOF) analysis were prepared first by dissolving 5 mg of sample powder in 10 mL of a 50:50 *v*/*v* acetone/water solution. Then 10 mg of this solution was added to 10 µL of a 2,5-dihydroxy benzoic acid (DHB) matrix. The locations dedicated to the samples on the analysis plaque were first covered with 2 µL of a NaCl solution 0.1 M in 2:1 *v*/*v* methanol/water, and predried. Then, 1 µL of the sample solution was placed on its dedicated location and the plaque was dried again. The reference substance used for the equipment calibration was red phosphorus.

MALDI-TOF spectra were obtained using an Axima-Performance mass spectrometer from Shimadzu Biotech (Kratos Analytical Shimadzu Europe Ltd., Manchester, UK) using a linear polarity-positive tuning mode. The measurements were carried out making 1000 profiles per sample with two shots accumulated per profile. The spectra precision is of +1 Da.

### 2.3. FTIR

Fourier Transform Infra-Red (FTIR) analysis were carried out using a Shimadzu IR Affinity-1 (Shimadzu Europe Ltd., Manchester, UK) spectrophotometer. A blank sample tablet of potassium bromide, ACS reagent from ACROS Organics (Geel, Belgium), was prepared for the reference spectra. Similar tablets were prepared by mixing potassium bromide with 5% weight on weight of the sample powders to analyze. The spectra were obtained in transmittance by combining 32 scans with a resolution of 2.0 cm^−1^ in the 400–4000 cm^−1^ range.

### 2.4. Cross-Polarisation Magic Angle Spinning Nuclear Magnetic Resonance (CP-MAS ^13^C NMR) Spectra

The reaction mixtures of glucose and sucrose with dimethyl carbonate and of the reaction of the product obtained with hexamethylene diamine were analyzed by solid state CP MAS ^13^C NMR. Spectra were obtained on a Brüker AVANCE 400 MHz (Brüker, Billerica, MA, USA) spectrometer with a 4 mm probe at a frequency of 12 kHz. The pulse duration at 90° was 4.1 µs, with a contact time of 2 ms and a recycling delay of 4 s. Chemical shifts were determined relative to tetramethyl silane (TMS) used as a control. The spectra were accurate to 1 ppm. The spectra were run with the suppression of the spinning side bands.

### 2.5. Thermomechanical Analysis (TMA)

Resin A2 and B2 were tested by TMA on a Mettler 40 (Viroflay, France) thermomechanical analysis apparatus. Triplicate samples of two beech wood plies each 0.6 mm thick bonded with either A2 or B2 for a total sample dimensions of 21 mm × 6 mm × 1.15 mm were tested in non-isothermal mode between 40 and 220 °C at a heating rate of 10 °C/min in three points bending on a span of 18 mm exercising a force cycle of 12 s (6 s/6 s). The classical mechanics relation between force and deflection *E* = [*L*3/(4*bh*3)][Δ*F*/Δ*f*] allows the calculation of the Young’s modulus *E* for each case tested [30,33].

### 2.6. Wood Joints Bonding

**Scheme 1:**
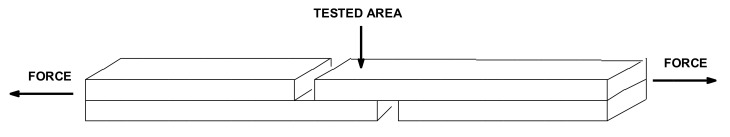
An exemplificative sketch of the bonded wood joint test.

Pine (*Pinus sylvestris*) wood veneers of 3 mm thickness were cut into boards of dimensions 7.5 cm × 18 cm. The adhesive-coated area for each veneer board was 2.5 cm × 18 cm. The glue spread used was of a 260 g/m^2^ double glue line, applied by a manual roller spreader, and after an open assembly time of 12 min and a closed assembly time of 10 min the joints were hot-pressed at a pressure of 2.75 N/mm^2^ and a temperature of 220 °C for 6 min to form a two-ply wood composite board. Three bonded joints were prepared with each adhesive combination. The joints were conditioned at ambient temperature and an equilibrium moisture content of 12%. Each bonded joint was cut into six samples with a bonded area of 2.5 cm × 2.5 cm. For each adhesive combination six samples were tested dry, six samples were immersed in cold water for 24 h then tested wet, and six specimen were immersed in boiling water for 2 h then cooled and tested wet, according to British Standard BS 1204 (1993) [34]. The tests were done in tension in an Instron 3300 dual-column universal testing machine (Instron France, Elancourt, France) at a head rate of 2 mm/min. The specimens were tested in tension, as shown in Scheme 1.

### 2.7. Surface Coatings Applications

Duplicate samples of both A2 and B2 were spread at a load of 150 g/m^2^ onto pine wood block surfaces 7.5 cm × 18 cm and were cured at 130 °C overnight (14 h) and at 300 °C for 5 min. The contact angles of a drop of water on the four coatings so prepared were monitored every one minute for 10 min and compared to the same for an untreated surface of wood. The contact angle of the treated and untreated wood surfaces was obtained from the water drop being placed on the surface with a syringe and measured with an EasyDrop contact angle apparatus, using drop shape analysis software (KrüsGmbH, Hamburg, Germany).

Equally, A2 and B2 were spread by knife on two stainless steel plates at a load of 50 g/m^2^, and then cured in an oven at 300 °C for 3 min. After cooling they were subjected to an adhesion test by the cross-cut test according to French (European) standard NF EN ISO 2409 [35]. For this, the coating was cut through to the metallic substrate with a razor blade in order to produce edges from which the coating may then be lifted. The cutting pattern consisted of a 10 × 10 grid of vertical and horizontal cuts spaced at 1 mm × 1 mm. A strong adhesive tape was then applied over the cut area and tightly pressed. The tape was then rapidly pulled off. This operation was repeated three times. Finally the coating was examined to determine the number of blocks removed, this constituting an evaluation of the coating adhesion.

## 3. Results and Discussion

Table 1 and Figure 1, Figure 2, Figure 3, Figure 4, Figure 5, Figure 6, Figure 7, Figure 8, Figure 9, Figure 10, Figure 11 and Figure 12 report the MALDI-ToF results obtained for the A2 NIPU resin based on glucose. There are examples of glucose molecules just reacted with the dimethyl carbonate (which are present in abundance in the A1 spectra, not reported here) such as the species at 263.8 Da.

The subsequent initial reaction of the hexamethylene diamine with this type of species forms a urethane link, such as the species at 322.8 Da.

Many species of this type appear to be formed (Table 1) where the glucose has formed two or more urethanes on the same glucose moiety, as well as species presenting both dimethyl carbonate-linked and urethanes formed, such as the species at 378.8 Da

**Figure 4:**
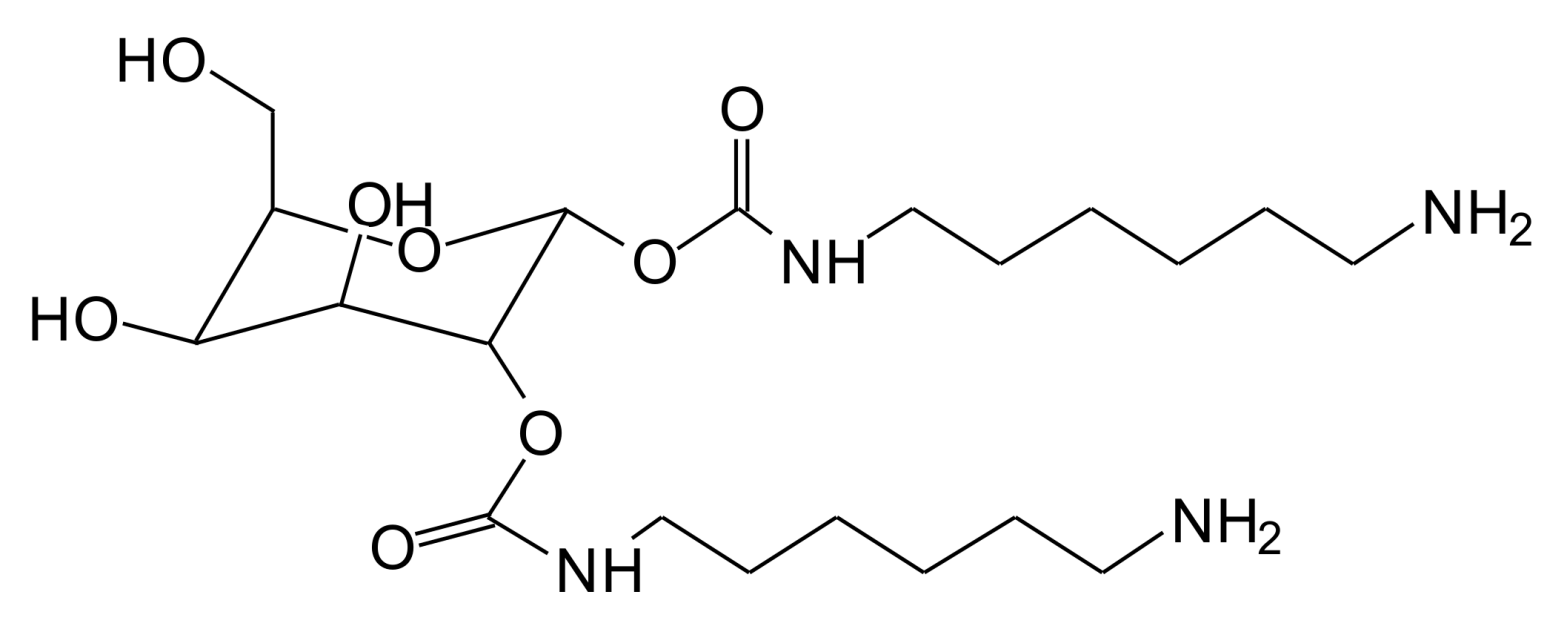
Example of one of the possible glucose diurethane structures.

**Figure 5:**
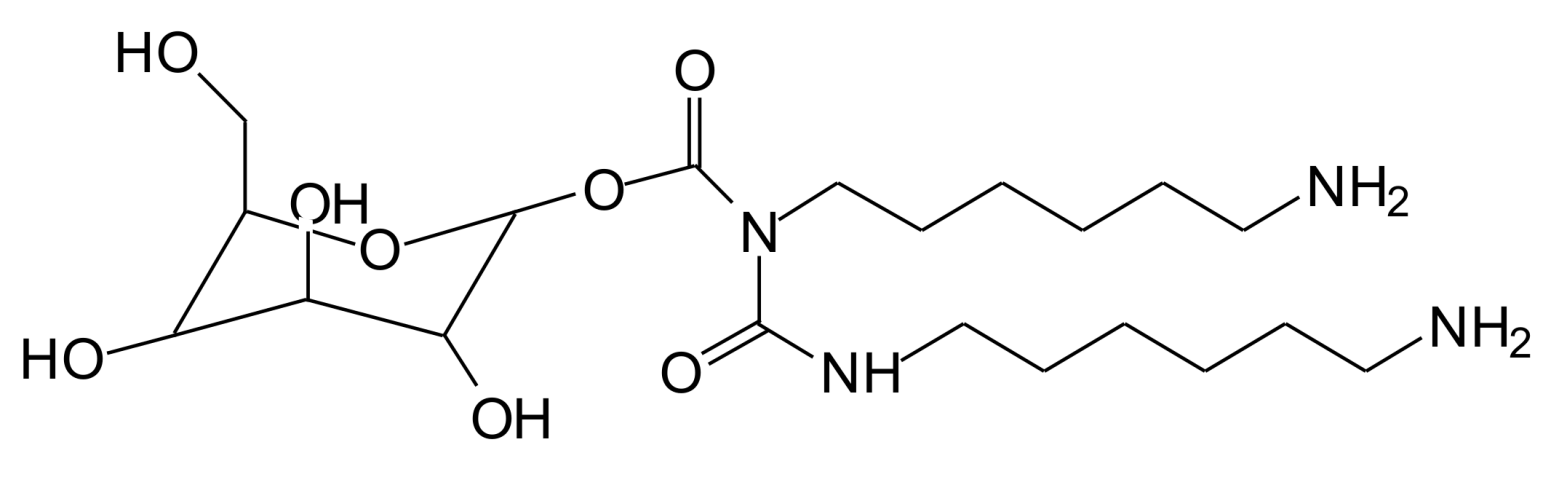
Example of alternative, possible glucose diurethane structures.

It must be clearly pointed out that for all the species indicated in Table 1, as shown in Figure 4:

The species can correspond also species of the same molecular weight but of structure as shown in Figure 5:

More interesting for the potential application of these NIPU resins are the oligomers formed, such as the trimer at 1225.6 Da, in which up to six urethane linkages are present, in its linear configuration.

Additionally, a multitude of possible branched configurations exist, such as:

The existence of branched species indicates that these resins do cross-link, as the results found, and are discussed later for both bonding and coating.

Of further interest are the results of CP MAS ^13^C NMR analysis of A1 carbonated glucose and of the A2 glucose-based non-isocyanate polyurethane resin shown in Figure 13 and Figure 14. These indicate that species other than those identified by MALDI ToF also occur in the reaction mixture.

The peak at 92.7 ppm in Figure 13 also attests that a number of species in which a glucosidic bond between two glucoses has been formed have also been formed such as the structure shown in Figure 15:

The peaks at 96.9 and 62.5 ppm belong to the glucose, the latter to the –CH_2_OH side chain of its pyranose ring. Of interest is the presence of the two peaks at 74.9–75 and 72.3 ppm. The former can be attributed to carbons of the glucose pyranose ring in both glucose moieties reacted and not reacted with DMC. The latter only appears for carbons belonging to carbonated glucose. A small peak at 26.7 ppm indicates that a very small proportion of glucose also appears to be present in its open form [36,37].

**Figure 7:**
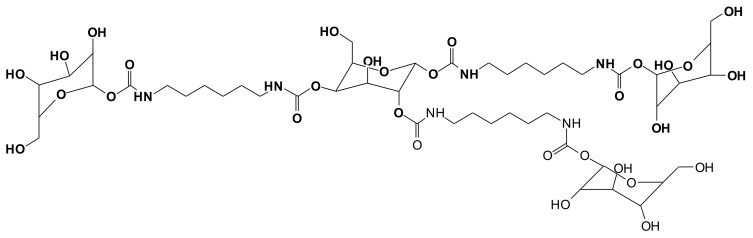
An example of one of the possible branched structures of the trimer at 1225.6 Da.

More complex, but more interesting, is the CP MAS ^13^C NMR of the final A2 resin shown in Figure 7. The interpretation of the peaks has been carried out according to literature references [31,32,36,37,38,39,40,41]. The peak at 61 ppm is the one belonging to the –CH_2_OH side chain of the pyranose ring. The peak is very small, but its presence indicates that a very small proportion of this glucose site has not reacted. The peak at 52 ppm is attributed to the methyl of the –COO–CH_3_ of carbonated glucose not reacted with the diamine, confirming the existence of species already identified by MALDI ToF. The peaks at 42 and 27.5–28 ppm belong to the methylene groups (–CH_2_–) of the diamine chain, respectively, directly linked and not linked to urethane groups. The 42 ppm thus confirms the reaction of the diamine and formation of urethane groups. The peak at 158 ppm is the peak belonging to the C=O of the –NH–COO– of the urethane bridges. The peak is quite marked indicating that the proportion of urethane formed is relatively high, in line with that indicated by the intensity of the 42 ppm peak. Up to here the signals remarked were expected, but even more interesting are the rest of the peaks. The very small peaks at 176 and 172 ppm could either belong to the formation of –COOH groups derived either from oxidation of the aldehyde group of the open form of glucose, or by a rearrangement of the dimethyl carbonate, although this latter appears unlikely. The peak at 164 ppm is due to an internal rearrangement giving the group. The most likely rearrangement is the one corresponding to a group of the type (Figure 16):

This indicates the possibility of very small amounts of oligomers formed by just the reaction of DMC and diamine. The very small peaks at 164, 129, and 108 ppm are indicative of internal rearrangements of the amine and of the amine-DMC adducts, rearrangements of the type shown in Figure 17.

These indicate perhaps some form of degradation of some products, although its extent is rather small. Finally, the peaks at 12.3 and 9.9 ppm are attributed to –CH_3_ groups in a sterically-hindered configuration. This latter probably belongs to the CH_3_CH_2_COOH or, even more probably, CH_3_CH_2_CONH_2_ formed by internal rearrangement of DMC or of the carbonated amine to an amide. These rearrangements are, however, small and do not appear to interfere with the performance of the material in both bonding and coatings applications.

**Figure 8:**
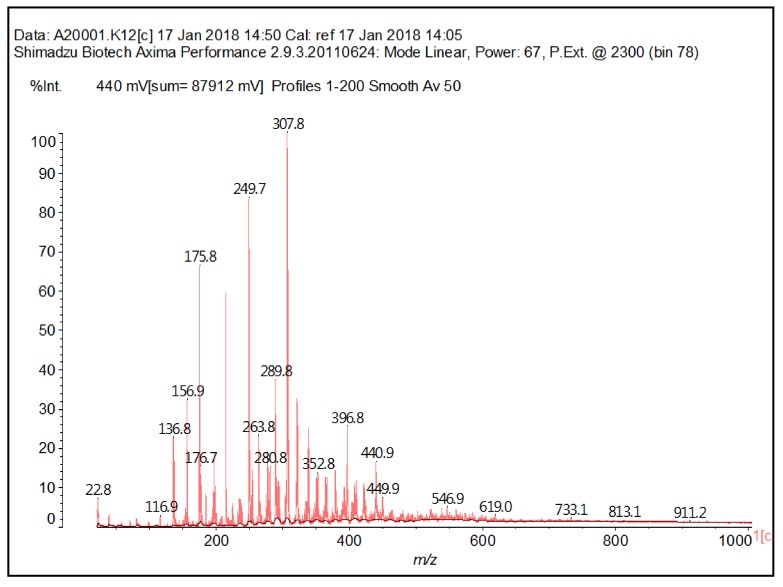
MALDI ToF spectrum of the A2 NIPU resin: 20–1000 Da range.

**Figure 13:**
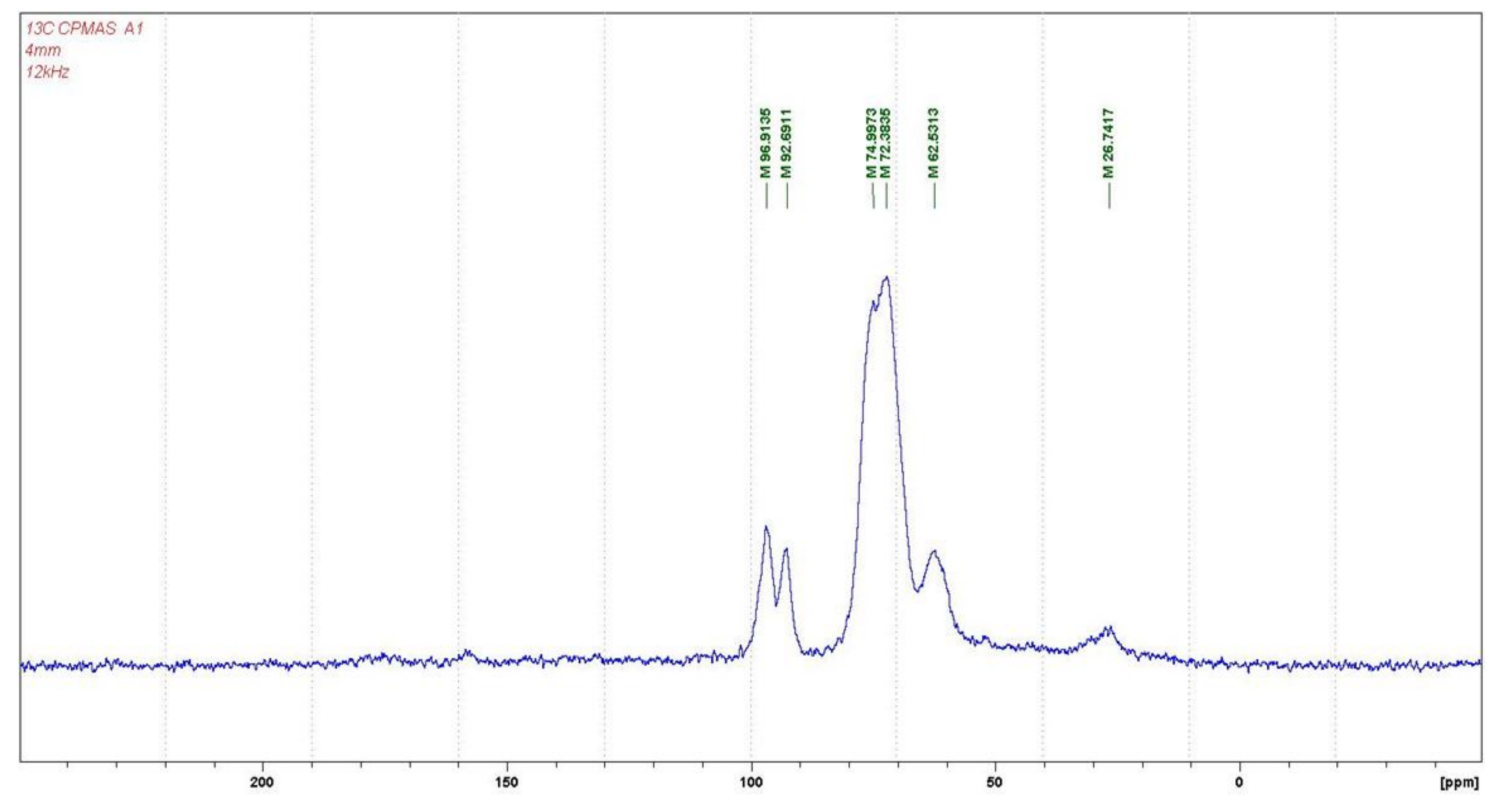
CP-MAS ^13^C NMR spectrum of the carbonated glucose A1.

**Figure 14:**
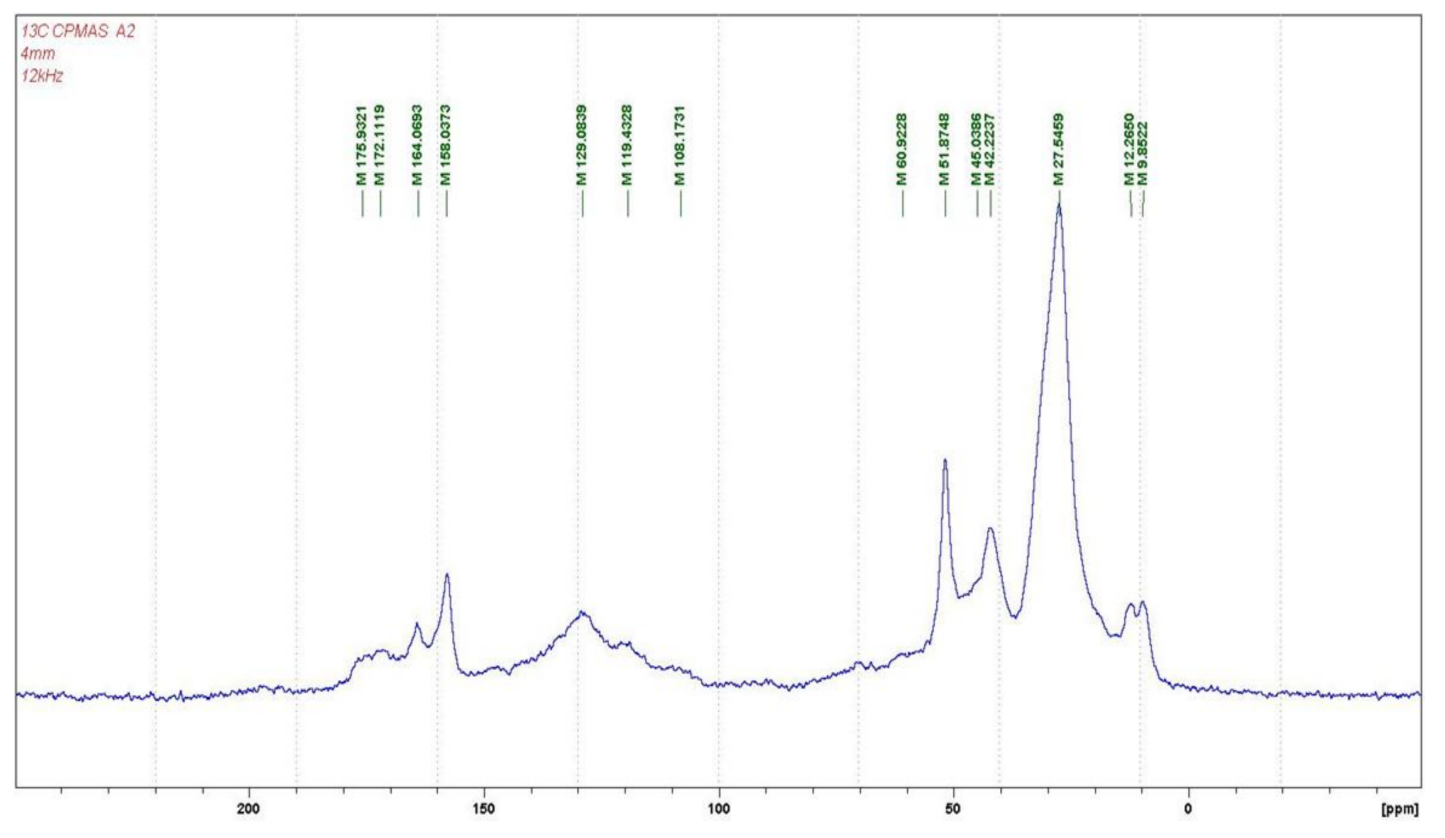
CP-MAS ^13^C NMR spectrum of the A2 NIPU resin.

**Figure 15:**
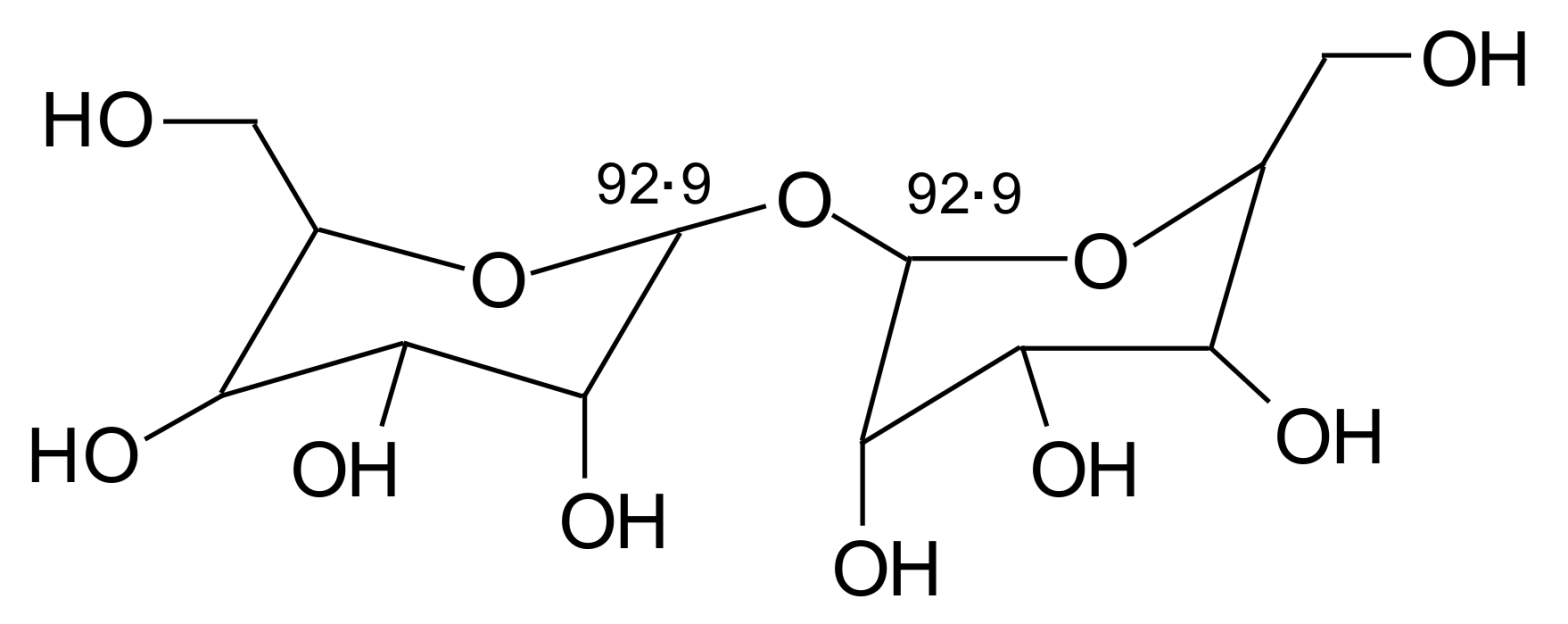
Example of two glucoses linked by a glucosidic bond formed by the elimination of CO_2_ from two glucoses linked by a dimethyl carbonate. The peak is shown in Figure 13.

**Figure 16:**
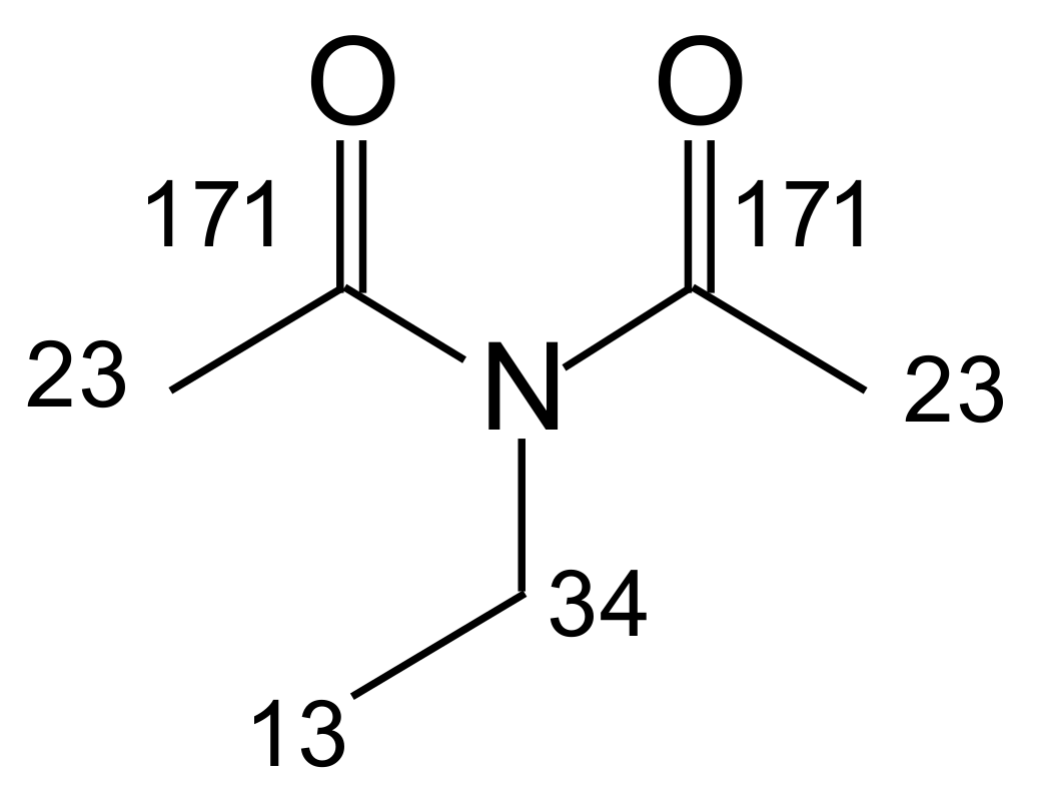
Example of rearranged group corresponding to the 164 ppm peak.

**Figure 17:**
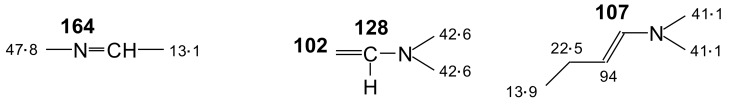
Internal rearrangement of the amine and the amine-DMC adducts corresponding to the 164, 129, 108 ppm peaks.

**Figure 18:**
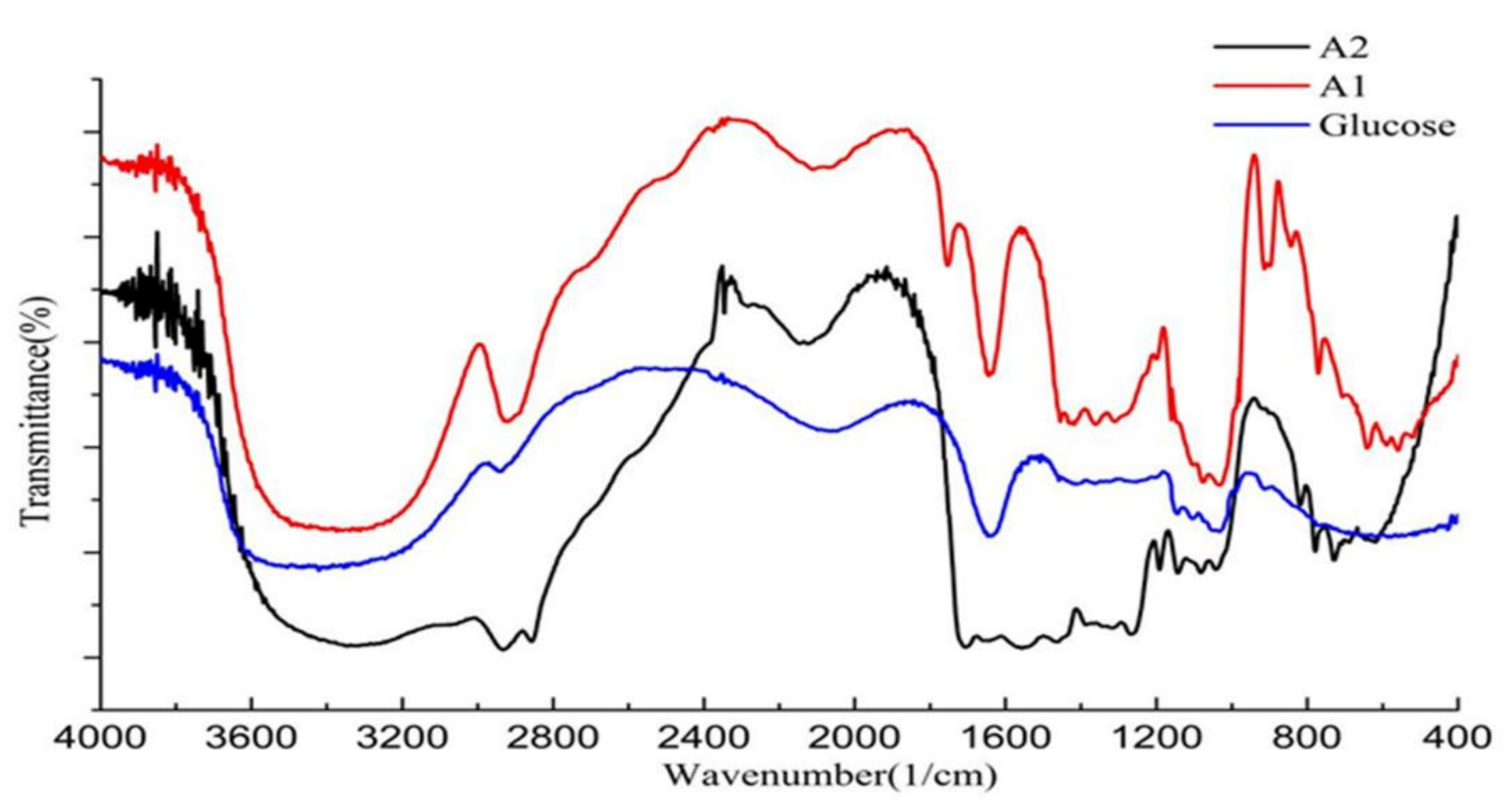
FTIR spectra of glucose, carbonated glucose A1, and glucose-based A2 NIPU resin.

**Figure 19:**
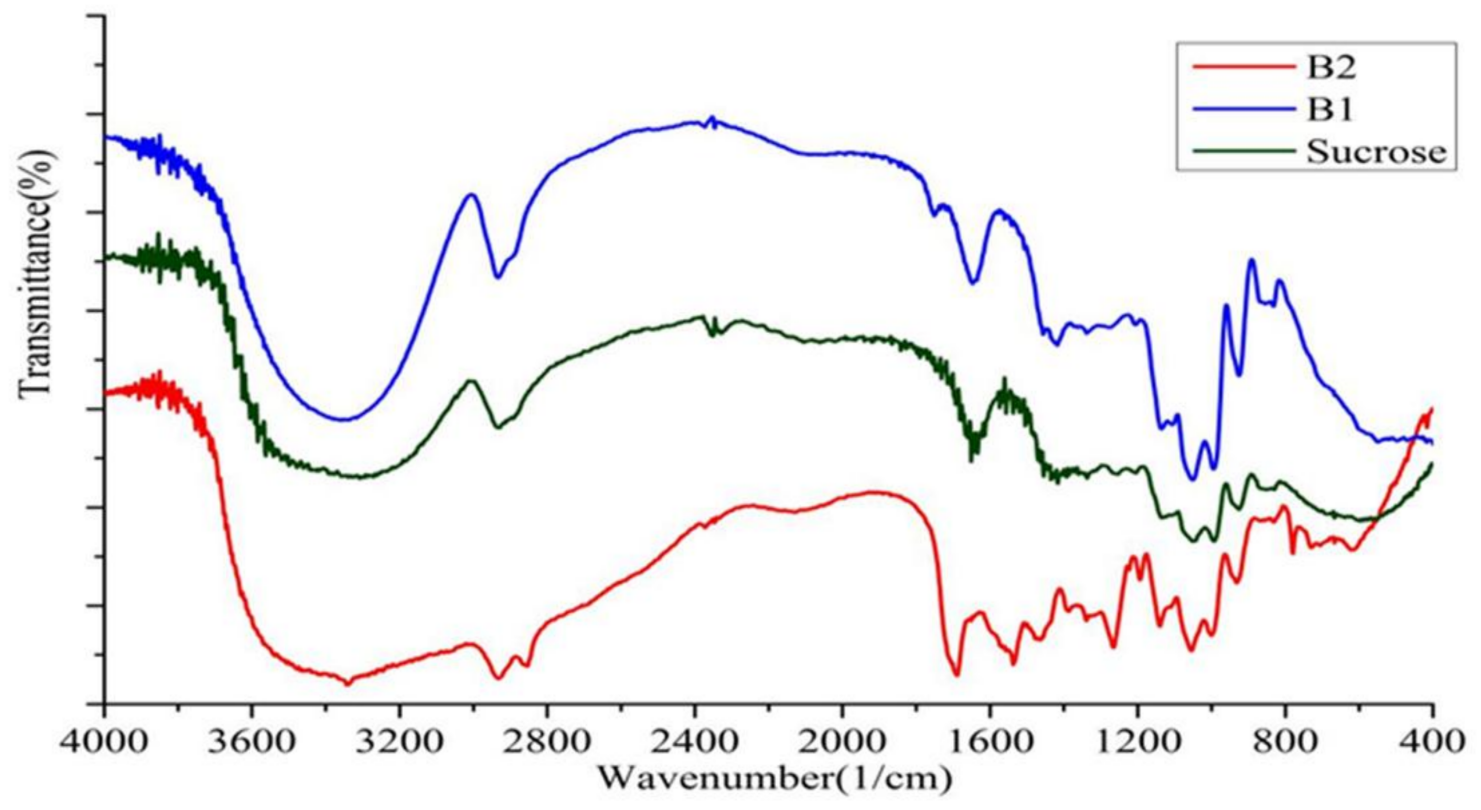
FTIR spectra of sucrose, carbonated sucrose B1, and sucrose-based B2 NIPU resin.

The results of the FTIR analysis of the A2 and B2 resins are shown in Figure 18 and Figure 19. The peaks for urethane linkages are seen at 1711, 1547, 1475, and 1251 cm^−1^ [39,40,41]. These are small and not so clearly seen. On the A1 FTIR in Figure 8 the 1744 cm^−1^ of the C=O of the carbonate and a peak at 1621 cm^−1^, that can possibly be attributed to the aldehyde group of the open form of the glucose, are very clearly visible. Table 2 reports the main IR peak assignments.

The specimens as shown in Scheme 1 were tested in tension. The results of bonded wood joints in Table 3 indicated that bonding did occur with the non-isocyanate glucose and sucrose polyurethane (NIPU) resins prepared. Those obtained from glucose appeared to be somewhat better and also easier to handle and to spread. These are, indeed, only initial exploratory results and the formulation and both ratios of reagents and the reaction process can be improved much further. However, it can be noted that, in the case of the A2-bonded joints, very interesting results are obtained after cold water soaking for 24 and 2 h in boiling water. These results are comparable to those obtained dry. Considering the liophilicity of glucose towards water this is remarkable and again confirms the formation of water repellent cross-linked polyurethane resins. One problem encountered is that the classical hardeners/accelerators used to form and harden polyurethanes are of no use in NIPU systems due to the absence of isocyanates. This means that these systems can harden exclusively by heat. Thus, cross-linking accelerators based on alternative reactions need to be found for this type of polyurethane obtained by such an alternative route. It must be clearly pointed out that A1 ad B1 are just intermediate products of the two-step reaction process, and it is for this reason that they have not been tested either for bonding or for coatings.

**Figure 20:**
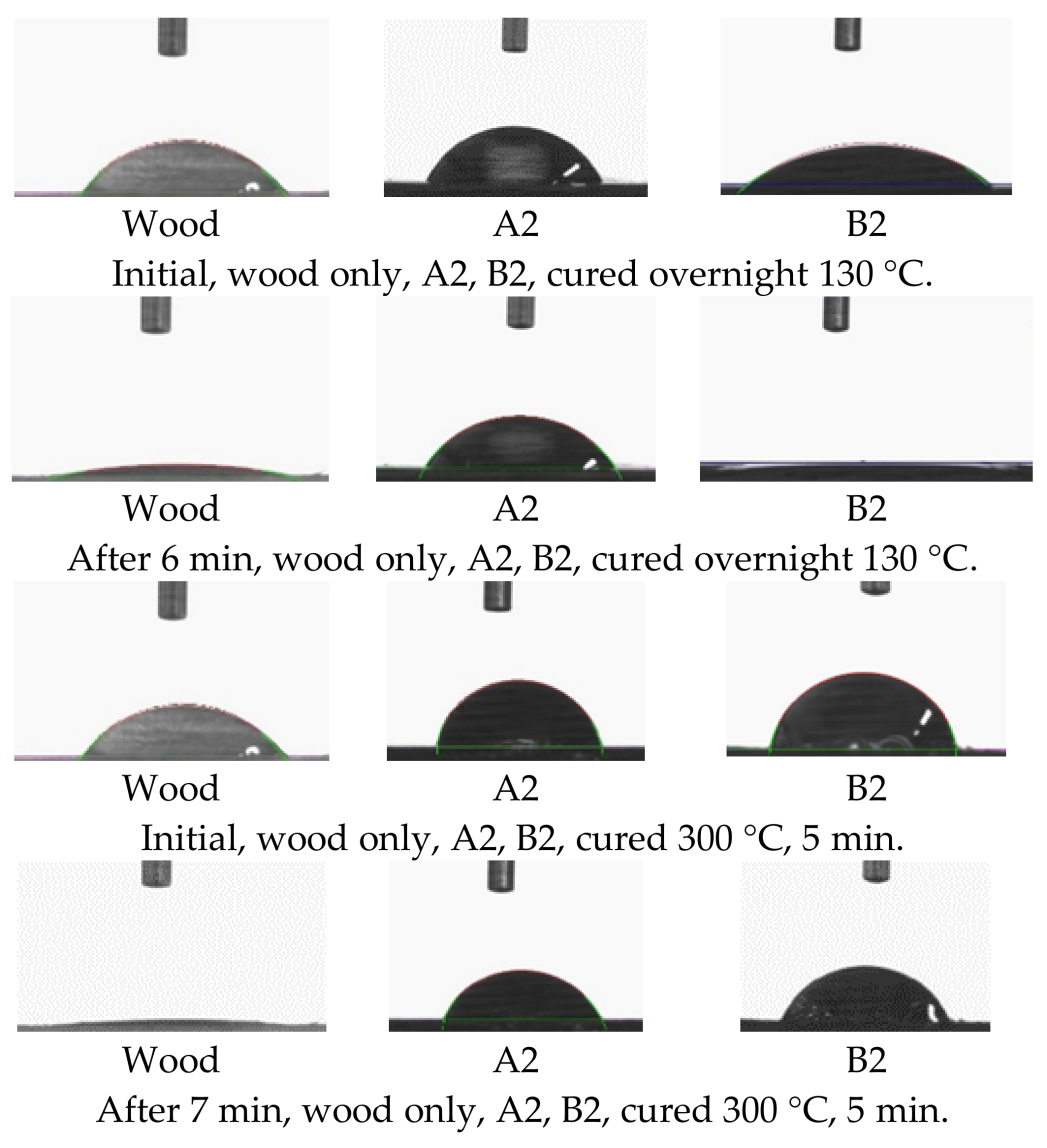
Comparative sessile water drop contact angles on an untreated wood surface, on a wood surface treated with a glucose-based A2 coating, and on a wood surface treated with a sucrose-based B2 coating. The initial angle when cured overnight at 130 °C (**top**). The angle after 6 min wetting when cured overnight at 130 °C (**second from Top**). The initial angle when cured at 300 °C for 5 min (**second from Bottom**). The angle after 7 min wetting when cured at 300 °C for 5 min (**bottom**).

Of considerable interest are the results obtained by coating wood surfaces with A2 and B2 NIPU resins. Thus, the contact angles of A2 coatings are always higher than those of both untreated wood and of B2 coated wood (Table 4 and Table 5; Figure 20). The contact angle for un-coated and coated wood are affected by the roughness of the contact surface and, thus, the results cannot surpass 90°.

The contact angles of water sessile drops on any wood surfaces tend to decrease with contact time. This is true since the tendency for a given mass of liquid to spread on a solid surface increases as the contact angle decreases [42,43,44,45,46,47]. 

**Figure 21:**
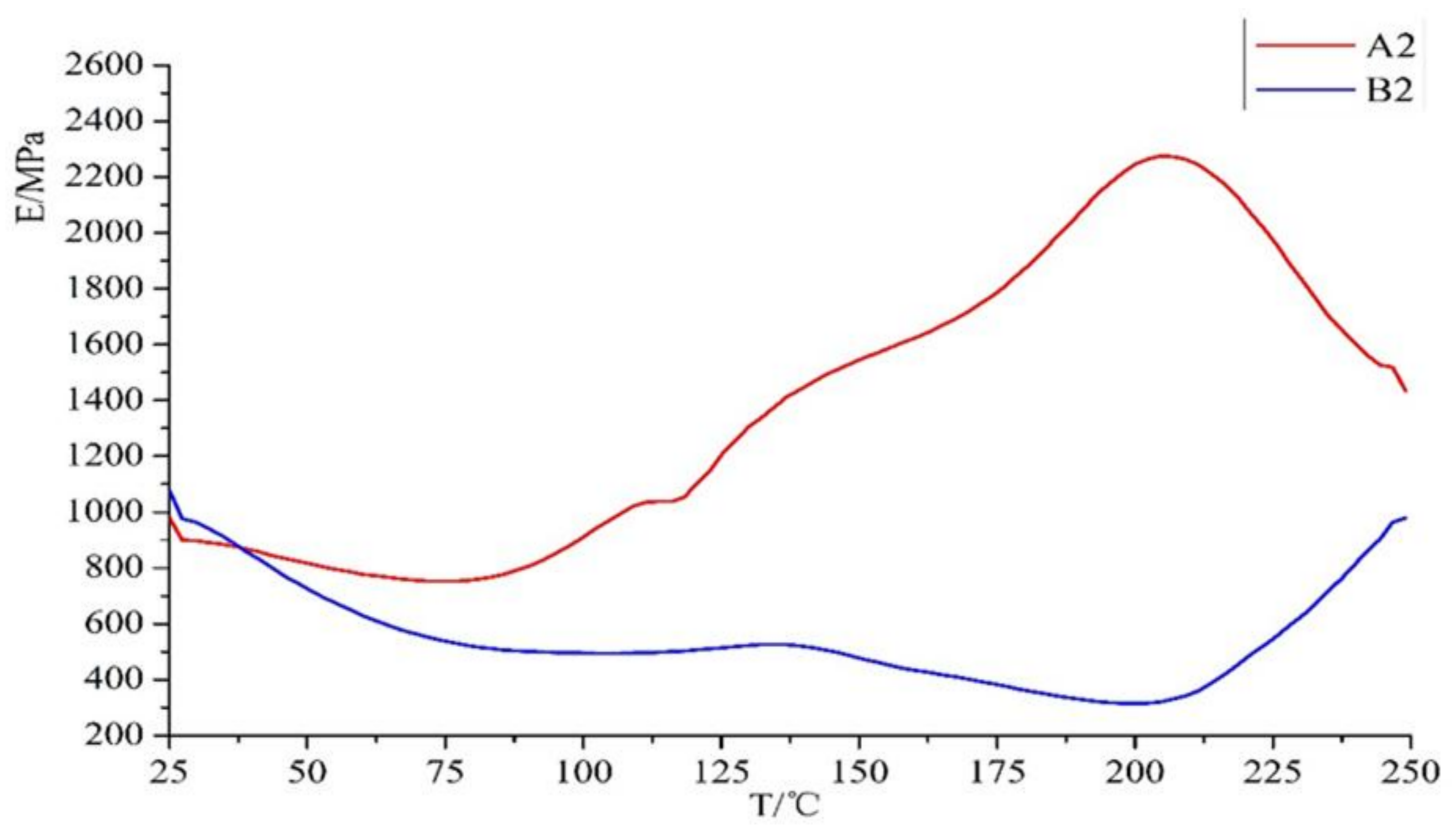
Comparative themomechanical analysis (TMA) traces of curing of A2 and B2 NIPU resins on wood.

TMA was used to prove that the curing temperature for A2 is lower than for B2. TMA is now frequently used in this manner to determine the differences in wood adhesives, and there are many references on this [33,43]. The TMA curves indicate the reason why A2 showed better bonding strength and better performance as a wood coating than B2. This infers that A2 coatings have the added advantage on B2 coatings that their energy of activation of hardening is apparently lower. This appears to be the case as at 130 °C A2 coatings are capable of cross-linking and hardening while B2 coatings are patently not able to (Table 4). This is confirmed by the thermomechanical analysis trace [42] shown in Figure 21 where A2 NIPU resin curing and cross-linking appears to starts at a much lower temperature than for the B2 NIPU resin. The A2 resin curing appears to start at around 100 °C and it reaches its max cure at around 200 °C, while the B2 NIPU resin only appears to start curing at 205 °C. These TMA results appear to indicate that for sucrose the problem may be due either to diffusion problems or to a higher energy of activation of hardening, or to both. The TMA is used here to monitor the progress of cross-linking and hardening of the resins [33,42]. As condensation and cross-linking proceeds the reaction slows down when one progressively approaches *T*_g_ (∞) due to difficulties in molecular diffusion in the medium [48,49]. This is possibly due to the higher viscosity of the sucrose based resin. A temperature of 300 °C, albeit for a short period of five minutes, is sufficient to cross-link, harden, and render water-repellent B2 coatings, also further improving the performance of A2 coatings, to the point that their level of water repellence is comparable (Table 5). It is in the field of wood surface coatings that mono- and di-saccharide based NIPU show the initial best promise.

**Figure 22:**
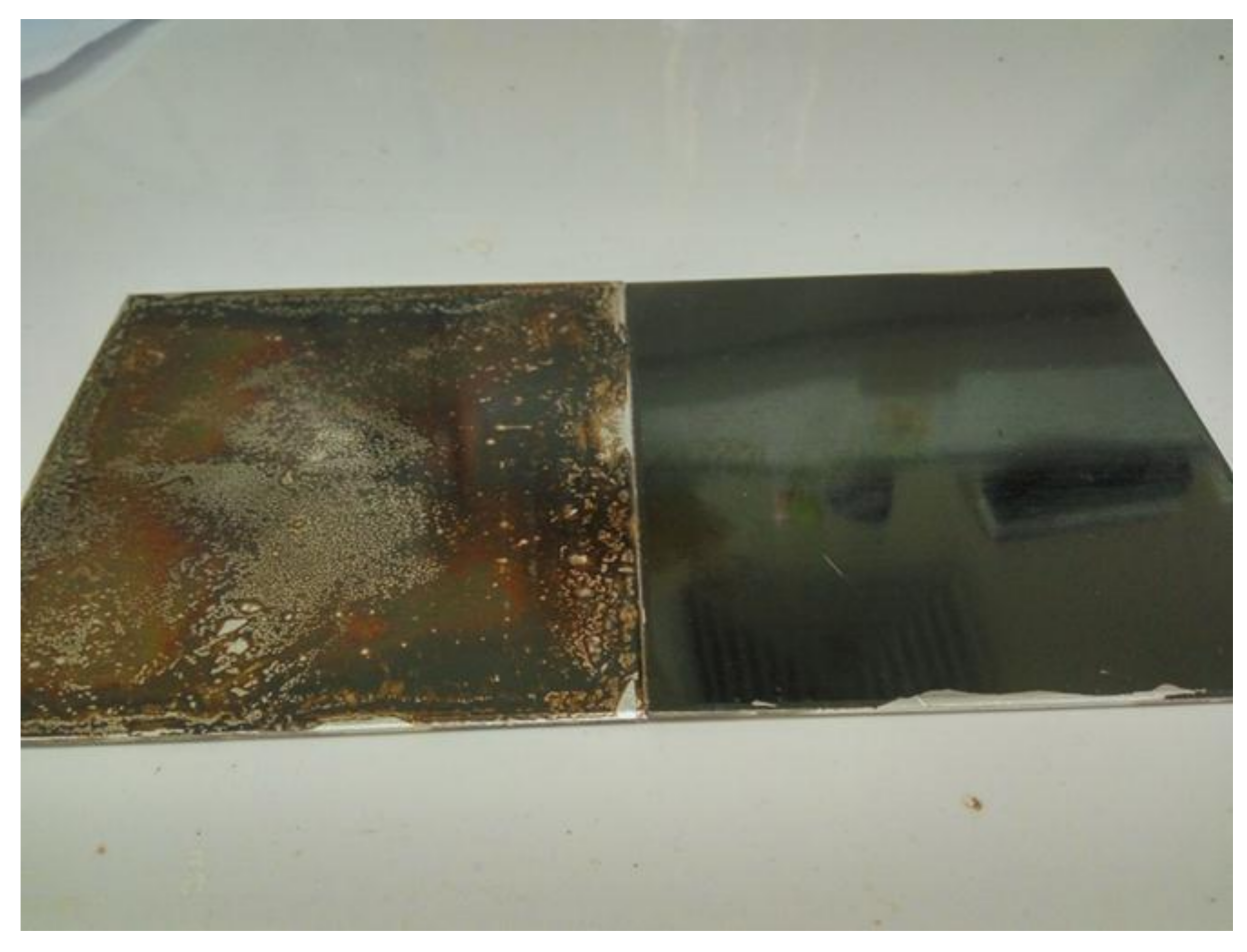
B2 (**left**) and A2 (**right**) NIPU resin surface coatings on stainless steel plates after curing at 300 °C for 3 min.

**Figure 23:**
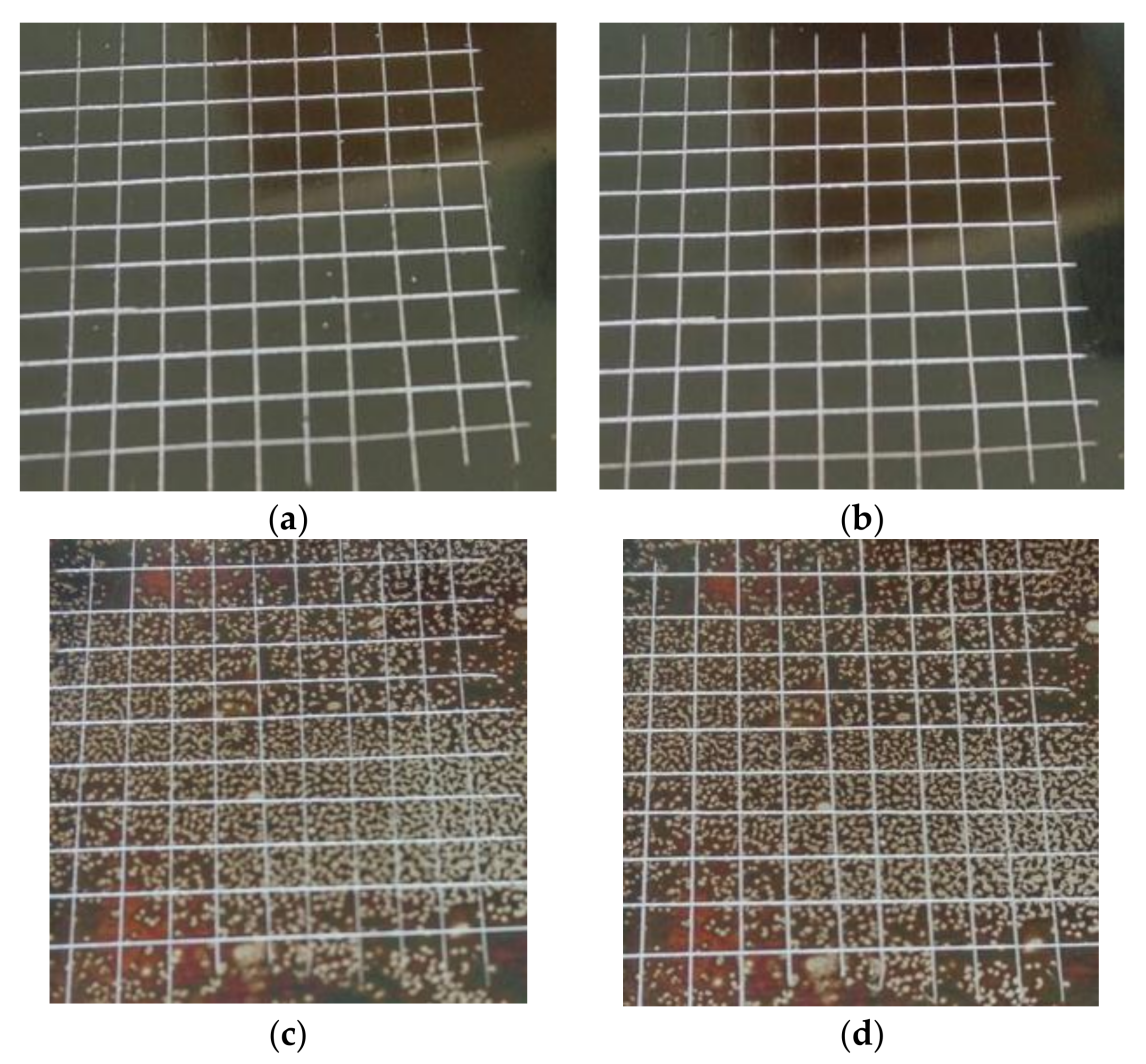
Cross-cut test according to NF EN ISO 2409 of glucose-based and sucrose-based surface coatings on stainless steel cured for 3 min at 300 °C. (**a**) A2 before washing in hot water; (**b**) A2 after washing in hot water; (**c**) B2 before washing in hot water; and (**d**) B2 after washing in hot water.

However, the potential of this type of NIPU resin is also shown by its capability of coating a steel surface. Figure 22 shows two steel plates coated with, respectively, B2 and A2. The figure shows clearly the better ease of spreading and good film appearance obtained with A2 coating on steel in relation to the poor performance of B2 coatings on steel. Of particular interest is the cross-cut adhesion test [34], the results of which are shown in Figure 23. This figure shows the aspect of the coated metal surfaces after the cross-cut test. None of the squares of the lattice are detached and the edges of the cut lines are smooth. Accordingly, adhesion to the metal plates of these coatings can be evaluated as being excellent. This is particularly true for coating A2 based on glucose, which presents an impressive performance.

## 4. Conclusions

Mono- and disaccharides appeared to yield workable non-isocyanate polyurethanes having a definite potential for application as both wood and steel surface thermosetting coatings and for thermosetting wood adhesives. Several type of oligomer species were observed by using different analytical techniques, namely by MALDI-ToF, ^13^C NMR, and FTIR. The products obtained were tested as surface coatings on both wood and steel, as well as to prepare bonded wood joints. The results obtained for bonded wood joints yielded encouraging results, although these will have to be further evaluated on the light of existing standards. The more interesting results were obtained for the application of glucose-based NIPU as wood surface coatings. These yielded high water wetting angles and with the wetting angle maintaining a high value as a function of time even at the lower temperature of curing used. In this respect glucose-based NIPU wood surface coatings appeared to perform better than sucrose-based NIPU. This is most likely based on a number of different reasons such as better ease of spreading and lower temperature of curing. At a higher curing temperature instead, the contact angles obtained for glucose- and sucrose-based wood coatings were comparable. This indicates that, for sucrose, the problem may be due either to diffusional problems or to a higher energy of activation of hardening, or to both. Again, for steel surface coatings, glucose-based NIPU performed much better than sucrose-based ones as regards their uniformity of surface spread. The cross-cut test indicated excellent resistance of these surfaces to mechanical wear. 

The main aim of this research was to show that NIPU can be prepared from mono- and disaccharides, this just being an initial exploratory work and its sole main aim. Mono- and disaccharides constitute a considerable reservoir of reasonably low-cost biosourced material easily available everywhere in the world rendering their use for such applications an interesting industrial alternative to existing routes of NIPU resins.

## Figures and Tables

**Figure 1:**
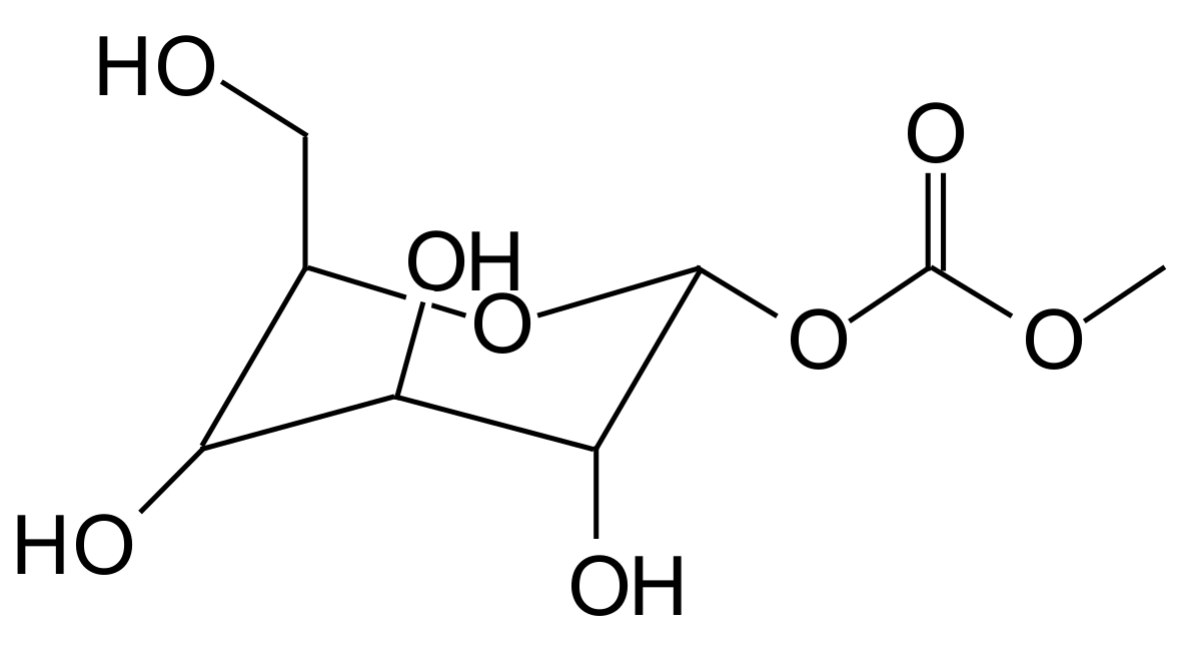
Monocarbonated glucose, peak at 263.8 Da.

**Figure 2:**
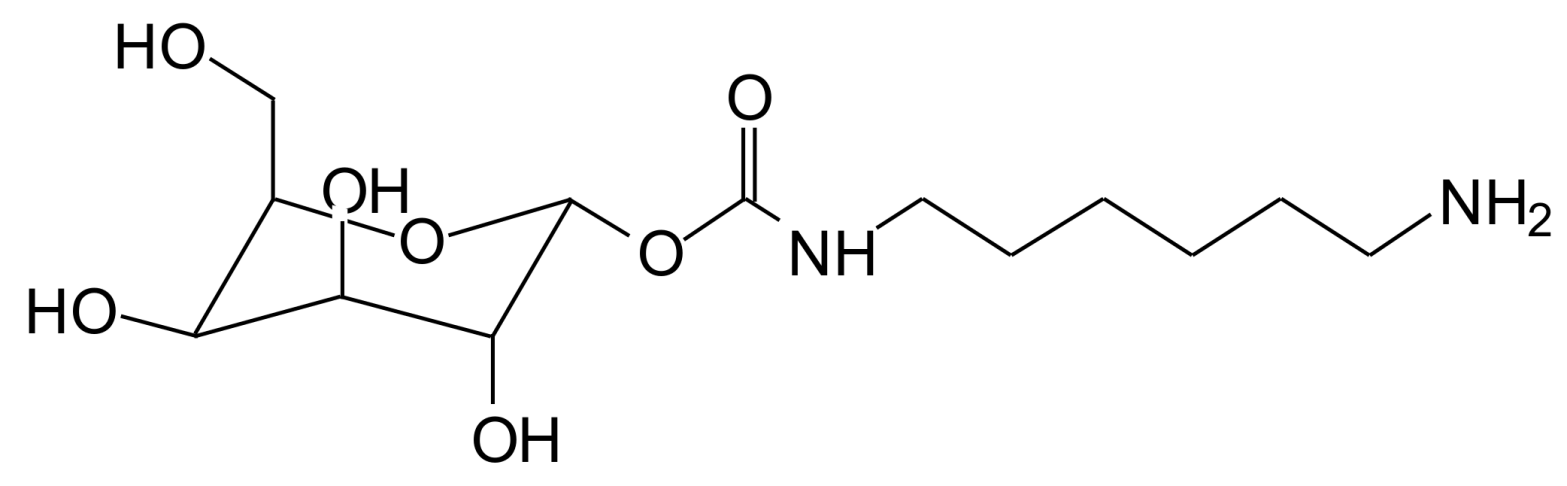
Urethane formed by reaction of monocarbonated glucose with hexamethylene diamine; 322 Da peak.

**Figure 3:**
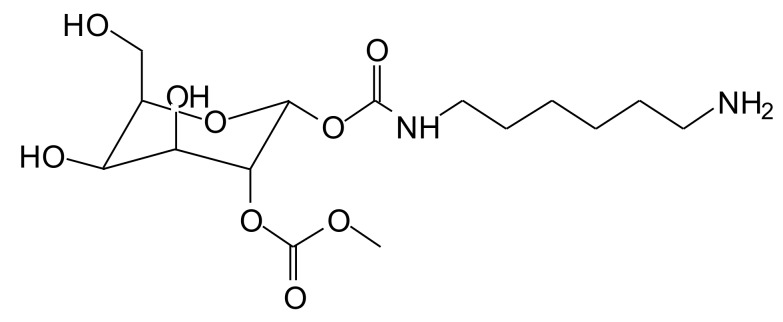
Example of a dicarbonated glucose having formed a urethane with hexamethylene diamine. 378.8 Da peak.

**Figure 6:**
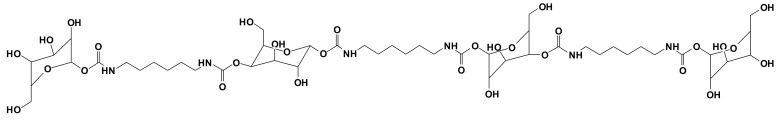
Example of the linear structure, one of the possible structures of the trimer at 1225.6 Da.

**Figure 9:**
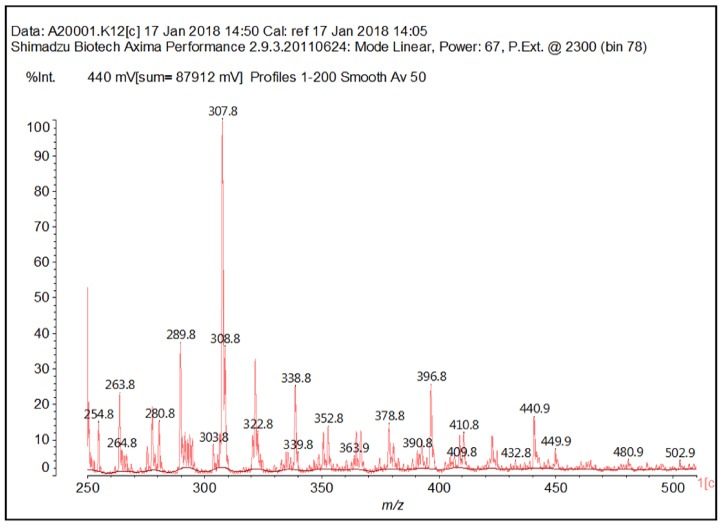
MALDI ToF spectrum of the A2 NIPU. Detail of the 250–500 Da range.

**Figure 10:**
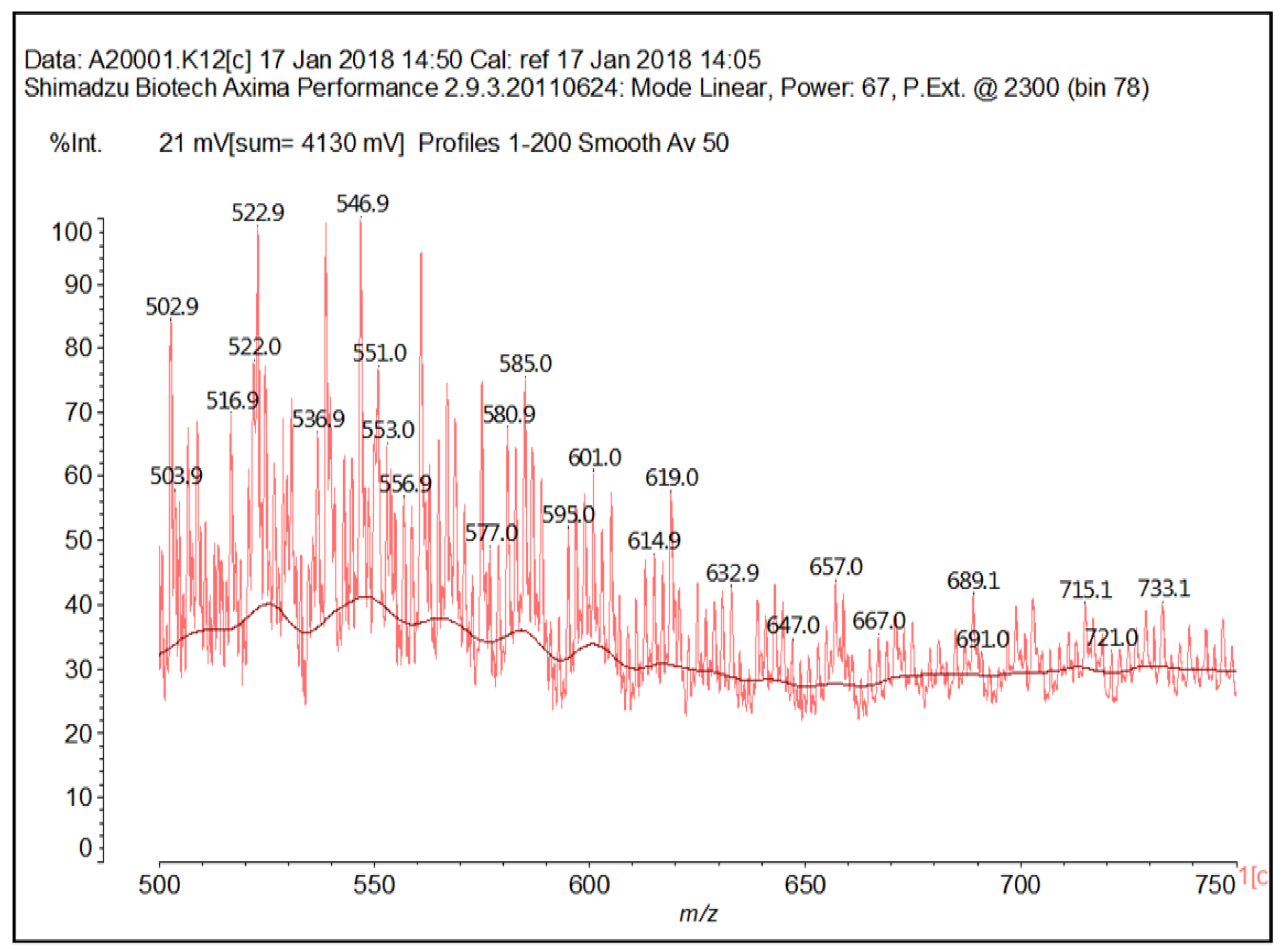
MALDI ToF spectrum of the A2 NIPU resin. Detail of the 500–7500 Da range.

**Figure 11:**
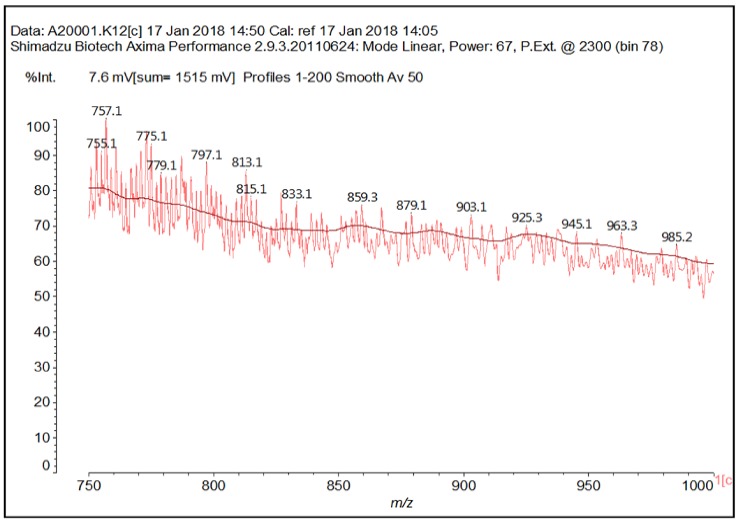
MALDI ToF spectrum of the A2 NIPU resin. Detail of the 750–1000 Da range.

**Figure 12:**
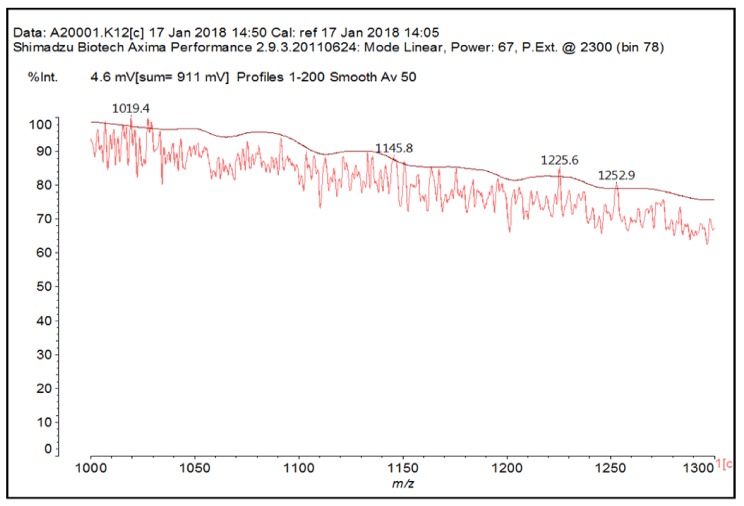
MALDI ToF spectrum of the A2 NIPU resin. Detail of the 1000–1300 Da range.

**Table 1:** Proposed oligomer structures for the MALDI ToF spectra peaks in Figure 1, Figure 2, Figure 3 and Figure 4 for the reaction of carbonated glucose with a diamine.

116.9 Da = dimethyl carbonate + Na^+^ 176.7 Da = glucose (calc. 180) deprotonated × 2 200 Da = glucose deprotonated + 23 Da (Na^+^) 263.8 Da (calc. 261 Da) (with Na^+^) protonated 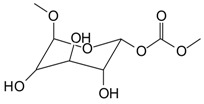 322.8 Da without Na^+^ 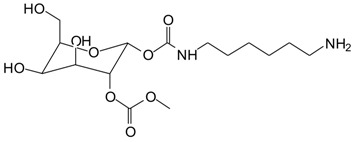 378.8 Da (calc. 380) without Na^+^ 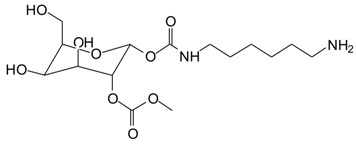 440.9 Da (Calc. 439) without Na^+^ 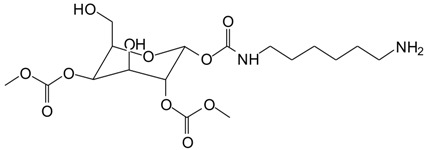 464 Da = without Na^+^ 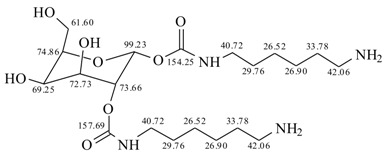 522 Da = 464 + 1 × DMC without Na^+^ 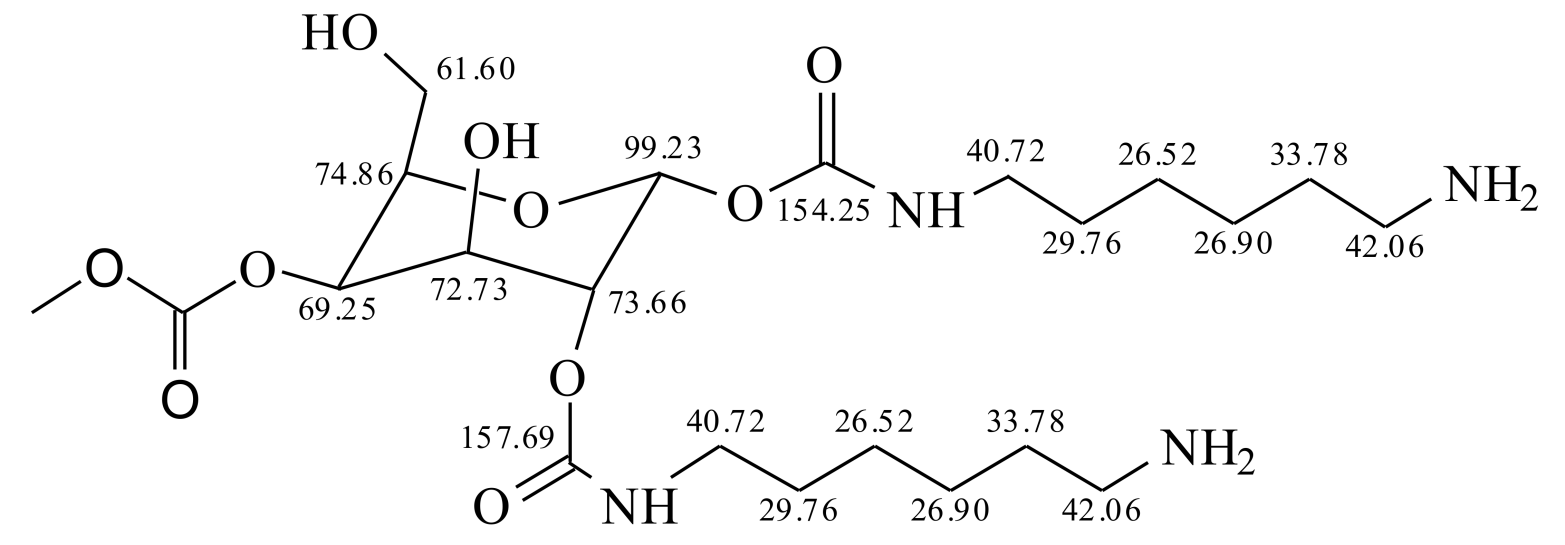 546 Da = 522 + 23 (Na^+^)580 Da = without Na^+^ 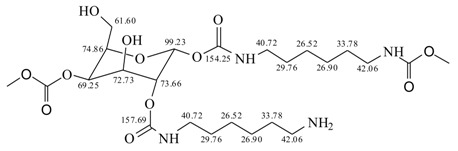 and/or 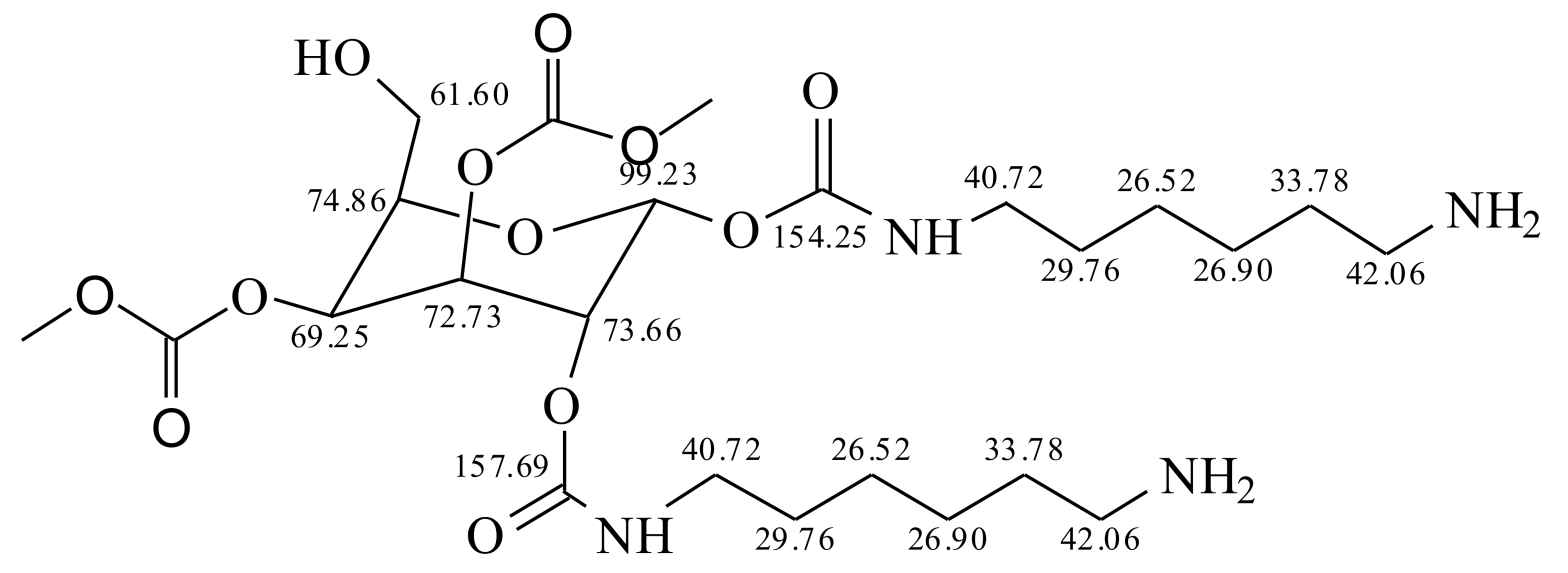 601 Da = 580 + 23 (Na^+^) deprotonated = (603 Da calculated) 632 Da (calc. 629) with + 23 Da (Na^+^) 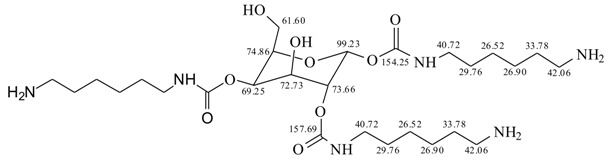 and/or 632.9 (calc. 635) without Na^+^ 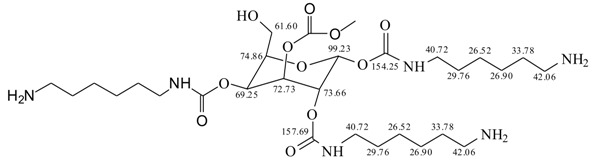 667 Da (calc. 675) with + 23 Da (Na^+^) 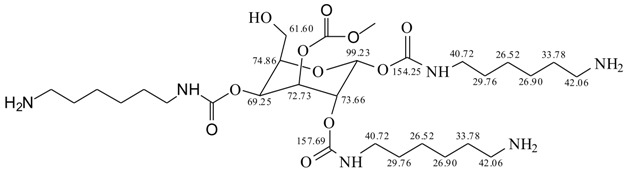 689 Da = 667 + Na^+^. Thus a case of a structure with 2 × Na^+^ 733 Da (730 calculated) without Na^+^ 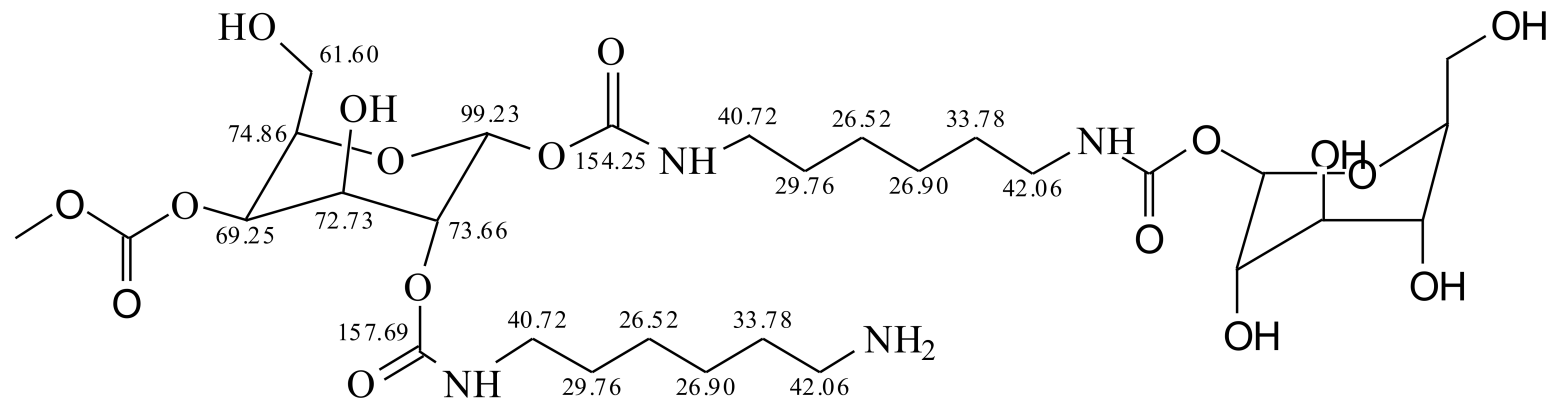 879 Da, without Na^+^ (calc. 878 Da) a PUR tetramer 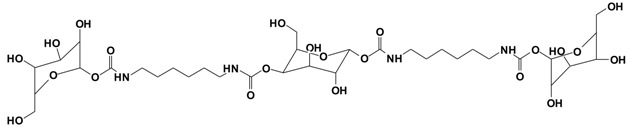 903 Da = 879 Da + Na^+^ (+23 Da) (Calculated 902 Da) a PUR tetramer 925 Da = 879 Da + 2× Na^+^ (calculated 926 Da) a PUR tetramer 1225.6 Da (1225.2 calculated), without Na^+^ (6 urethane linkages) PUR tetramer 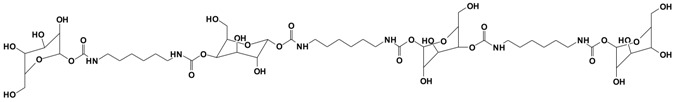 **And** 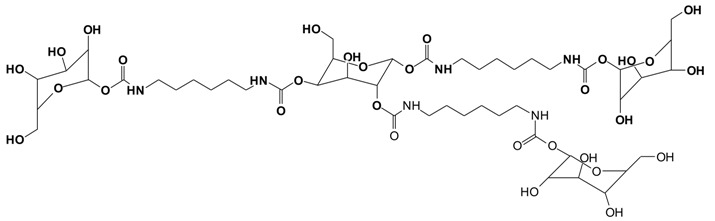

**Table 2:** FTIR peak assignments.

Peaks (cm^−1^)	Assignment
1051, 1087, 1150	C–O–C stretching vibration
1193	C–O stretching vibration
1325	–C–N stretching vibration
1394.5	–CH3 Surface bending vibration
1573	N–H bending vibration, urethane bridge
1642	C=O (carbonyl) stretching vibration
1718	C=O stretching vibration of urethane bridge
1744	C=O stretching of carbonate
2857, 2932	C–H stretching vibration
3100–3200	N–H, O–H stretching vibration

**Table 3:** Strength results of wood joints bonded with glucose (A2) and sucrose (B2) NIPU resins.

Resin	Dry strength (MPa)	24 h Cold Water Soak Strength (MPa)	2 h Boiled Strength (MPa)
A2	3.15 ± 0.05	3.62 ± 0.02	3.38 ± 0.04
B2	2.76 ± 0.09	1.32 ± 0.08	1.24 ± 0.04

**Table 4:** Water contact angle theta on glucose (A2) and sucrose (B2) NIPU resins cured at 130 °C overnight.

	Wood	A2	B2
Time (Minutes)	Theta (Degrees)	Theta (Degrees)	Theta (Degrees)
0	49.1 ± 1.24	64.4 ± 1.41	40.0 ± 2.17
1	36.1 ± 0.43	63.2 ± 1.30	20.6 ± 3.22
2	31.0 ± 1.68	61.6 ± 1.64	11.2 ± 0.10
3	28.2 ± 2.06	61.0 ± 1.19	8.8 ± 0.0
4	22.6 ± 1.66	59.9 ± 1.36	5.3 ± 0.39
5	19.5 ± 3.24	58.3 ± 1.35	3.6 ± 0.0
6	13.6 ± 0.81	57.2 ± 1.34	-
7	-	56.9 ± 0.97	-
8	-	54.7 ± 1.95	-
9	-	50.6 ± 1.02	-
10	-	49.2 ± 0.97	-

**Table 5:** Water contact angle on glucose (A2) and sucrose (B2) NIPU resins cured at 300 °C for 3 min.

	A2	B2
Time (Minutes)	Theta (Degrees)	Theta (Degrees)
0	80.6 ± 1.82	80.3 ± 0.65
1	76.3 ± 2.56	79.0 ± 1.40
2	74.5 ± 2.18	77.2 ± 2.15
3	73.4 ± 2.54	75.4 ± 2.04
4	72.1 ± 2.05	73.4 ± 0.38
5	69.0 ± 2.31	73.5 ± 0.78
6	67.7 ± 2.04	71.4 ± 1.70
7	65.9 ± 1.44	68.8 ± 1.70
8	64.8 ± 2.22	67.9 ± 2.81
9	62.9 ± 1.19	66.3 ± 2.69
10	62.3 ± 1.37	64.7 ± 1.70
